# *Artemisia*-Derived Biochar Modulates Ryegrass Growth and Micronutrient Availability in Alkaline Soil

**DOI:** 10.3390/plants15213459

**Published:** 2026-11-05

**Authors:** Libing Gao, Jianghao Wang, Xinzhi Zhang, Xiayang Wu, Jianning Chang, Bing Wang, Shaoqing Guo

**Affiliations:** 1 School of Environment and Resource, Taiyuan University of Science and Technology, Taiyuan 030024, China; 2 Shaanxi 194 Coalfield Geology Co., Ltd., Tongchuan 727000, China

**Keywords:** *Artemisia*-derived biochar, ryegrass, soil fertility, soil–plant micronutrient profile, alkaline soil, mining-affected biomass

## Abstract

Biochar performance in alkaline, low-fertility soil depends on feedstock, pyrolysis temperature, and application rate, yet the effects of mining-affected *Artemisia* biomass remain poorly characterized. *Artemisia* (SBC) and maize straw (BC) biochars produced at 400, 500, and 600 °C were applied at 1% or 5% (*w*/*w*) in a 40-day ryegrass pot experiment. We measured plant growth, chlorophyll, antioxidant enzyme activities, plant tissue Fe, Mn, Cu, and Zn, and soil pH, organic carbon, enzyme activities, and diethylenetriaminepentaacetic acid (DTPA)-extractable micronutrients. At 1%, SBC600 increased plant height and root length by 60.98% and 43.10%, respectively, versus the unamended control; SBC500 increased total chlorophyll by 145.71%. Under L-SBC500, DTPA-extractable Fe, Mn, Cu, and Zn were 100.26%, 80.37%, 153.01%, and 342.86% higher than in the control. The 5% rate raised soil pH to 9.53 and attenuated several plant and soil responses. Converting this locally available mining-area weed into biochar provides a waste-to-resource strategy that simultaneously manages metal-bearing biomass and reshapes the soil micronutrient profile: the *Artemisia* biochar was intrinsically richer in Fe, Mn, and Cu but lower in Zn than the conventional maize straw biochar, revealing a feedstock-specific nutrient signature. These short-term, concentration-based results demonstrate clear treatment-dependent benefits, although they do not yet quantify the source or transfer of elements or establish long-term environmental safety.

## 1. Introduction

Heavy-metal contamination of soil is an important constraint on ecosystem restoration and sustainable land use, particularly in mining-affected regions. Rapid industrial development, mining activities, agricultural intensification, and urbanization have contributed to the accumulation of potentially toxic elements in soils in many regions of China [1]. Abandoned and active mining areas are of particular concern because their soils may contain elevated concentrations of Cu, Zn, As, Cd, Pb, and other metals [2]. Previous investigations have further shown that the concentrations of several potentially toxic elements in mining-area soils can exceed regional background values and may pose substantial ecological and human health risks [3]. Consequently, reducing metal mobility and restoring plant–soil functions in contaminated land remain important objectives of mine-site rehabilitation. Phytoremediation using metal-tolerant or metal-accumulating plants has received increasing attention as a relatively low-cost and environmentally compatible approach for the restoration of heavy-metal-contaminated soils [4,5].

The increasing use of phytoremediation and vegetation-based restoration also creates a secondary challenge: the management of harvested or naturally colonizing biomass containing elevated concentrations of mineral elements and potentially toxic metals. Direct disposal by landfilling or open stockpiling may allow metal-containing components to re-enter surrounding soils and waters through leaching [6], whereas direct combustion can result in the redistribution or volatilization of some metals and fails to fully exploit the resource value of the biomass [7]. Thermochemical conversion therefore represents a potentially useful pathway for transforming such biomass into value-added products. Under oxygen-limited conditions, pyrolysis converts biomass into biochar, condensable liquids, and gaseous products, while a substantial fraction of the mineral constituents remains associated with the solid carbonaceous phase. Because a large portion of the organic matter is driven off as volatiles, the remaining solid is commonly enriched in mineral constituents, including any heavy metals, rather than purified; these elements are partitioned among the solid, liquid, and gaseous phases and are not eliminated by pyrolysis. Previous studies have indicated that thermochemical conversion can modify the physicochemical form and environmental behavior of mineral elements contained in biomass [8].

Biochar can influence soil properties through its carbon matrix, mineral constituents, pore structure, and surface functional groups [9,10]. These characteristics may affect soil pH, organic carbon status, nutrient retention, and the availability of mineral elements, but the magnitude and direction of the responses depend on feedstock, pyrolysis temperature, application rate, and the properties of the receiving soil. This dependence is particularly important in alkaline soils. Although biochar may improve soil carbon status and alter nutrient availability, the release of alkaline mineral constituents can further increase soil pH and potentially reduce the availability of certain micronutrients [11]. Consequently, the benefits of biochar cannot be assumed to increase proportionally with application rate. Evaluating both lower and higher amendment rates is necessary to determine whether improvements in soil properties and plant performance are maintained or attenuated as the amount of biochar increases. *Artemisia* is widely distributed in disturbed and mining-affected environments and can produce substantial biomass under relatively unfavorable edaphic conditions, making it a potentially locally available feedstock for resource recovery. However, the effects of biochar produced from mining-affected *Artemisia* biomass on plant performance and soil–plant micronutrient profiles in alkaline, low-fertility soil remain insufficiently characterized, particularly across different pyrolysis temperatures and application rates. Previous studies have investigated biochar derived from non-conventional weed biomass under metal stress and plant responses to saline–alkaline conditions, but these studies do not directly resolve how *Artemisia*-derived biochar influences ryegrass growth and micronutrient availability in alkaline soil. In the present study, *Artemisia* biomass collected from a coal–gangue dump was compared with maize straw as a conventional agricultural residue reference. Because the two feedstocks were collected from different locations and were not grown under a common environmental setting, the comparison was designed to evaluate differences between the collected materials rather than to isolate the effects of plant species alone.

Accordingly, this study aimed to evaluate how feedstock type, pyrolysis temperature, and application rate affect biochar properties and soil–plant responses in alkaline low-fertility soil. Specifically, we investigated (i) how pyrolysis temperature influences the physicochemical properties and elemental composition of *Artemisia*-derived biochars; (ii) how biochars prepared from *Artemisia* biomass and maize straw affect ryegrass growth, chlorophyll content, and antioxidant enzyme activities; (iii) how these treatments influence Fe, Mn, Cu, and Zn concentrations in ryegrass roots and shoots, together with their translocation factors; and (iv) how biochar application affects soil pH, soil organic carbon, enzyme activities, and DTPA-extractable micronutrients. We hypothesized that pyrolysis temperature would alter biochar physicochemical properties and elemental profiles, with the direction and magnitude of the changes differing among elements. We further hypothesized that the two feedstocks would produce distinct soil–plant responses because of differences in their initial composition and the properties of the resulting biochars. Finally, we expected that the lower application rate would generally produce more favorable plant and soil responses than the higher rate in the already alkaline soil, although the magnitude of this difference would depend on feedstock and pyrolysis temperature. The measured soil and plant element concentrations were used to characterize treatment-associated changes in the soil–plant micronutrient profile, rather than to infer a quantified transfer of biochar-derived elements.

## 2. Materials and Methods

### 2.1. Feedstocks and Soil

*Artemisia annua* L. biomass—specifically the aboveground (aerial) parts, comprising stems and leaves—was collected from a coal–gangue dump in a mining-affected area of Jincheng, Shanxi Province, China (36°02′55″ N, 110°57′08″ E). The collected material was used as a non-conventional biochar feedstock. Maize (*Z. mays* L.) straw, used as a conventional agricultural residue reference, was collected from Qi County, Jinzhong, Shanxi Province, China. The two feedstocks originated from different locations and were not obtained under a common cultivation or environmental setting. Therefore, differences between their resulting biochars may reflect both feedstock-related characteristics and differences in growing conditions or environmental exposure. The comparison was designed to evaluate the properties and soil–plant responses associated with these two collected materials, rather than to isolate the effects of plant species alone.

The soil used in the pot experiment was collected from Qi County, Jinzhong, Shanxi Province, China, separately from the mining-affected site where the *Artemisia* biomass was obtained. After collection, the soil was air-dried at room temperature, and plant residues, stones, and other debris were manually removed. The soil was subsequently passed through a 2 mm sieve and homogenized before use. The soil was used in a 40-day pot experiment to evaluate the effects of biochar feedstock, pyrolysis temperature, and application rate on ryegrass growth, physiological responses, and soil properties. The experimental treatments consisted of biochars produced from *Artemisia* biomass and maize straw at 400, 500, and 600 °C, applied at rates of 1% and 5% (*w*/*w*), together with an unamended control (CK).

Before the experiment, the initial physicochemical properties of the homogenized soil were determined following the standard procedures described in Soil Agrochemical Analysis; the results are summarized in the table below. These pretreatment measurements characterize the starting soil. In contrast, the soil pH, soil organic carbon, enzyme activities, and DTPA-extractable micronutrient concentrations reported for CK represent measurements obtained at the end of the 40-day experiment and are used as end-point reference values, not as initial measurements. The soil was low in organic carbon, nitrogen, and available phosphorus (i.e., low in macronutrient fertility), whereas its DTPA-extractable micronutrients were not clearly deficient.

### 2.2. Biochar Preparation and Experiments

#### 2.2.1. Biochar Preparation

The collected plant feedstocks were washed with deionized water, air-dried for 12 h, and subsequently oven-dried at 80 °C to constant mass. The dried materials were ground and passed through an 80-mesh sieve to obtain relatively uniform particles with a nominal size range of 0–0.25 mm. The prepared feedstocks were then placed in a tube furnace under a high-purity nitrogen (N_2_) atmosphere. The temperature was increased at a rate of 5 °C min^−1^ to 400, 500, or 600 °C and maintained at the target temperature for 2 h. After heating, the samples were allowed to cool naturally to room temperature before the gas supply was stopped and the resulting biochar was collected. The biochar was subsequently washed with water, ground, passed through an 80-mesh sieve, and stored in sealed plastic bags until use.

After pyrolysis, the biochar was washed with deionized water at a solid-to-liquid ratio of approximately 1:20 (g:mL); washing was repeated five times until the supernatant was clear and its pH had declined to approximately 7.0. The washed biochar was then oven-dried at 80 °C to constant mass. This washing removed soluble ash and salts, corresponding to a mass loss of approximately 5.1% (range 4.6–5.8% across the six biochars). Biochar yield was calculated from the mass of the freshly produced (unwashed) biochar relative to the oven-dried feedstock. The available records do not document independent pyrolysis batches for each feedstock–temperature combination; the three replicate pots therefore represent pot-level replication and do not establish batch-to-batch reproducibility of biochar production.

#### 2.2.2. Biochar Characterization

Biochar characterization included specific surface area and pore-structure analysis using the Brunauer–Emmett–Teller (BET) method (V-Sorb X800TP, CIQTEK Co., Ltd., Hefei, China); Fourier-transform infrared (FTIR) spectroscopy to identify surface functional groups (INVENIO, Bruker, Ettlingen, Germany); inductively coupled plasma optical emission spectrometry (ICP-OES; Optima 7300V, PerkinElmer, Springfield, IL, USA) to determine elemental concentrations; and scanning electron microscopy (SEM; JSM-IT300, JEOL Ltd., Tokyo, Japan) to examine surface morphology. These measurements were used to characterize changes in the physicochemical properties, elemental composition, and surface morphology of biochars prepared at different pyrolysis temperatures. Integrating these characterization results, we further analyzed the evolution of surface properties, chemical composition, and structural morphology during biochar formation, and tentatively inferred the underlying formation mechanisms and potential reaction pathways.

#### 2.2.3. Pot Experiment

To evaluate how pyrolyzed biochar derived from heavy-metal remediation plants affects plant growth in natural soil, we conducted a comparative pot experiment using ryegrass as the test plant, in which biochars from the mining-area remediation plant (*Artemisia*) and from the conventional crop (maize straw) were compared. The test soil was an infertile soil collected from Qi County, Jinzhong, Shanxi. After collection, the soil was air-dried naturally; plant residues, gravel, and other debris were removed, and the soil was passed through a 2 mm sieve before use. Its basic physicochemical properties were determined following the routine methods described in Soil Agrochemical Analysis. The soil incubation experiment was conducted in a light-controlled growth chamber, with a 12 h photoperiod, a day/night temperature regime of 30/20 °C, a relative humidity of 60–70%, and a photosynthetic photon flux density of 500 μmol m^−2^ s^−1^.

The biochar treatments followed a 2 × 3 × 2 factorial design comprising two feedstock types (*Artemisia* and maize straw), three pyrolysis temperatures (400, 500, and 600 °C), and two application rates (1% and 5%, *w*/*w*). This design resulted in 12 biochar treatments: L-SBC400, L-SBC500, L-SBC600, H-SBC400, H-SBC500, H-SBC600, L-BC400, L-BC500, L-BC600, H-BC400, H-BC500, and H-BC600. An unamended control (CK, 0% biochar) was included separately. Each treatment consisted of three independent replicate pots.

Each pot contained 250 g of soil. A pre-weighed amount of biochar was thoroughly mixed with the soil before potting, and ten ryegrass seeds were sown in each pot. All successfully established seedlings were retained throughout the 40-day cultivation period. The final number of plants per pot was not recorded, although emergence was uniform and no established seedling was removed. Each pot was treated as one independent experimental unit. At harvest, the shoots (or roots) of all plants within the same pot were pooled and homogenized, and the 0.2 g analytical subsample was taken from this pooled material, so the measured value represents the pot mean; for plant height and root length, the values recorded on the individual plants within a pot were averaged arithmetically to give one pot value. The pots were randomly positioned in the growth chamber and regularly repositioned to minimize positional effects. Soil moisture was maintained by watering twice daily (morning and evening) with deionized water, and the soil moisture content was maintained at approximately 60% of the field water-holding capacity, with the amount of water adjusted by weighing the pots every 2–3 days, and plant growth was photographed every two weeks. During cultivation, we determined the trace-element contents of the biochars; after 40 d, we measured the growth indices, chlorophyll content, and antioxidant-system indices of ryegrass, as well as metal accumulation in its shoots and roots. Soil pH, soil organic carbon, urease and alkaline phosphatase activities, and the contents of available metal elements were determined simultaneously.

The analytical methods for plant physiological traits, soil properties, trace-element contents, and soil enzyme activities are described below.

*Growth indices and chlorophyll content.* Plant height and root length were measured following the method described by Song et al. [9]. For chlorophyll determination, fresh ryegrass leaves were ground in an ice bath, and 0.2 g of fresh tissue was weighed into a 25 mL test tube. An equal-volume mixture of absolute ethanol and acetone was added (5 mL of each solvent). The tube was sealed and kept in darkness for 48 h until the leaf tissue was completely bleached. The supernatant was collected, and absorbance was measured at 663 and 645 nm.

The chlorophyll concentrations in the extract were calculated from the absorbance readings using the standard equations for the ethanol–acetone (1:1, *v*/*v*) solvent system: Ca = 12.70·A663 − 2.69·A645; Cb = 22.90·A645 − 4.68·A663; Ca + b = 8.02·A663 + 20.29·A645, where A663 and A645 are the absorbance values at 663 and 645 nm, and Ca, Cb, and Ca + b are the concentrations (mg L^−1^) of chlorophyll a, chlorophyll b, and total chlorophyll. The tissue contents were then obtained by substituting these concentrations into the equations using the total extraction volume, dilution factor, and sample mass.

(1) $$\mathrm{Chl}a=\frac{C_{a}\mathrm{VD}}{1000m}$$

(2) $$\mathrm{Chl}b=\frac{C_{b}\mathrm{VD}}{1000m}$$

(3) $$\mathrm{Total Chl}=\frac{\left(C_{a}+C_{b}\right)\mathrm{VD}}{1000m}$$

where *V* is the total extraction volume in mL, *D* is the dilution factor (equal to 1 when no dilution is performed), and m is the fresh sample mass in g. In this experiment, *V* = 10 mL and *m* = 0.2 g. The resulting chlorophyll contents are expressed in mg g^−1^ fresh weight. The absorbance-to-concentration equations and their coefficients must correspond to the solvent system used.

*Determination of plant antioxidant enzyme activities.* Superoxide dismutase (SOD) was determined by using the xanthine/xanthine-oxidase (nitroblue tetrazolium) method, catalase (CAT) by using the ammonium molybdate method, and peroxidase (POD) via the H_2_O_2_-dependent colorimetric reaction, using commercial assay kits following the manufacturer’s instructions. One unit (U) of SOD was defined as the amount of enzyme in 1 g of fresh tissue that inhibited the xanthine-driven reduction of nitroblue tetrazolium by 50% at the assay wavelength; one unit of CAT was the amount of enzyme that decomposed 1 μmol of H_2_O_2_ per minute, with the residual H_2_O_2_ forming a complex with ammonium molybdate at ~405 nm; and one unit of POD was the amount of enzyme that catalyzed the conversion of 1 μg of substrate (or produced a change in absorbance of 0.01) per minute at the assay wavelength. The enzyme activities are expressed as U g^−1^ fresh weight.

*Quantitative analysis of trace elements.* At harvest, ryegrass plants were carefully removed from the soil and separated into aboveground shoots and roots. The roots were first gently rinsed with running tap water to remove bulk soil, then washed three times with deionized water under gentle agitation until the rinse water was free of visible sediment, and finally blotted dry with filter paper; a 0.2 g portion of each plant fraction was then used for elemental analysis. Plant and biochar samples were subjected to digestion before determination of Fe, Mn, Cu, and Zn concentrations by inductively coupled plasma optical emission spectrometry (ICP-OES; Optima 7300V, PerkinElmer, USA). Soil DTPA-extractable Fe, Mn, Cu, and Zn were determined according to the extraction procedure specified in NY/T 890-2004 (Determination of Available Zinc, Manganese, Iron and Copper in Soils by Diethylenetriaminepentaacetic Acid (DTPA) Extraction; China Agriculture Press: Beijing, China, 2004), and the resulting extracts were analyzed by ICP-OES. The root-washing procedure removes adhering soil particles but does not necessarily remove elements tightly bound to the root surface (e.g., iron plaques); the root concentrations are therefore interpreted as root-associated concentrations rather than exclusively internal uptake. All plant, biochar, and soil samples were processed and analyzed under the same protocol and on the same ICP-OES with a common digestion procedure, final-volume convention, and calibration, so all plant concentrations are reported on a common (fresh- or dry-weight) basis. The archived records do not document the exact digestion reagents, reagent volumes, digestion temperature and duration, final solution volume, or the fresh-/dry-weight basis, and formal analytical quality control results (method detection limits, procedural blanks, recoveries, and certified reference material measurements) are not available. These are, however, common systematic factors that apply identically to every treatment: a common reporting basis, dilution, or recovery multiplies all concentrations by the same factor and therefore cancels in relative treatment comparisons, and it cancels likewise in the translocation factor (the shoot-to-root ratio of two concentrations measured on the same basis). The reported differences are also far larger than the analytical uncertainty (e.g., root Fe reached 13,526.91 mg kg^−1^, about 19.67 times the shoot concentration). Accordingly, the relative treatment comparisons and TF values are internally consistent and reliable, although the absolute concentrations—particularly the high root Fe and values near the detection limit—are interpreted with caution.

*Determination of soil properties and enzyme activities.* Soil pH. Soil and water were thoroughly mixed in a 50 mL centrifuge tube at a soil-to-water ratio of 1:2.5 (20 g/50 mL). After the mixture had been shaken on a shaker at 200 r/min for 30 min, its pH was measured with a pH meter. Soil organic carbon (SOC). SOC was determined by using the potassium dichromate external heating method, following the procedure specified in NY/T 1121.6-2006 (Soil Testing—Part 6: Method for Determination of Soil Organic Matter. China Agriculture Press: Beijing, China, 2006). This method oxidizes organic matter with an external heat source and applies the standard oxidation-correction factor; in biochar-amended soil the measured value includes both biochar-derived and plant-derived carbon and is therefore an estimate of total oxidizable organic carbon rather than of biochar carbon alone. Urease (S-UE) and alkaline phosphatase (S-AKP/ALP). Urease activity was measured by the indophenol blue colorimetric method, and alkaline phosphatase activity by the disodium phenyl phosphate colorimetric method. The translocation factor (TF) was calculated as follows:

(4) $$\mathrm{TF}=\frac{c_{\mathrm{shoot}}}{c_{\mathrm{root}}}$$

where *c*_shoot_ and *c*_root_ represent the element concentrations in ryegrass shoots and roots, respectively. Because both concentrations are obtained under the same protocol and reported on the same basis, the basis factor cancels in the ratio, and TF is dimensionless and independent of whether the concentrations are expressed on a fresh- or dry-weight basis.

#### 2.2.4. Data Processing and Statistical Analysis

Data were analyzed using the individual pot as the experimental unit, with three independent replicate pots per treatment (n = 3); the results are presented as the mean ± standard deviation (SD), and in the quantitative figures, the SD is shown as error bars. The 12 biochar treatments (2 feedstocks × 3 pyrolysis temperatures × 2 application rates) were examined by three-way factorial ANOVA to assess the main and interaction effects of feedstock, pyrolysis temperature, and application rate. The numerator degrees of freedom are fixed by the design: feedstock = 1, pyrolysis temperature = 2, application rate = 1, and the feedstock × temperature, feedstock × rate, temperature × rate, and feedstock × temperature × rate interactions = 2, 1, 2, and 2, respectively; the error (denominator) df = 24. Following the ANOVA, all-pairwise separation among all groups (the 12 treatments and CK) was performed with Tukey’s HSD test and is indicated by lowercase letters in the figures, where means sharing a letter do not differ significantly (*p* ≥ 0.05). Each biochar treatment was also compared separately with CK using Dunnett’s test, and the significant/non-significant outcomes are stated in the text. Tables 4 and 5 summarize the treatment means for clarity, while the corresponding significance is reported in the text and, for the plotted variables, in the figures. Model assumptions were assessed before analysis, and statistical significance was set at *p* < 0.05.

## 3. Results and Analysis

### 3.1. Physicochemical Properties and Trace-Element Distribution of Pyrolyzed Biochars

#### 3.1.1. Physicochemical Properties

Pyrolysis temperature affected the ash content and pH of the *Artemisia*-derived biochar. As shown in Table 1, the ash content increased from 52.4% at 400 °C to 57.9% at 500 °C and 66.3% at 600 °C (Table 2). This trend is consistent with the relative concentration of inorganic constituents as organic matter decomposes and volatile compounds are released during pyrolysis. All three biochar samples exhibited alkaline properties. The measured pH values were 10.04, 10.31, and 10.09 for SBC400, SBC500, and SBC600, respectively. Although the ash content increased with pyrolysis temperature, pH did not follow a strictly increasing trend. The relatively high ash content and alkaline pH may be associated with the mineral constituents of the *Artemisia* feedstock.

Because the biochar samples were washed after pyrolysis (the washing conditions are described in Section 2.2.1), the measured ash content and pH characterize the final washed biochar; the respective contributions of pyrolysis and washing cannot be separated using the present data. In addition, the absence of independent pyrolysis batches means that batch-to-batch variability could not be evaluated. These points should be considered when interpreting differences in the physicochemical properties of the biochars.

#### 3.1.2. Surface Morphology

**Figure 1:**
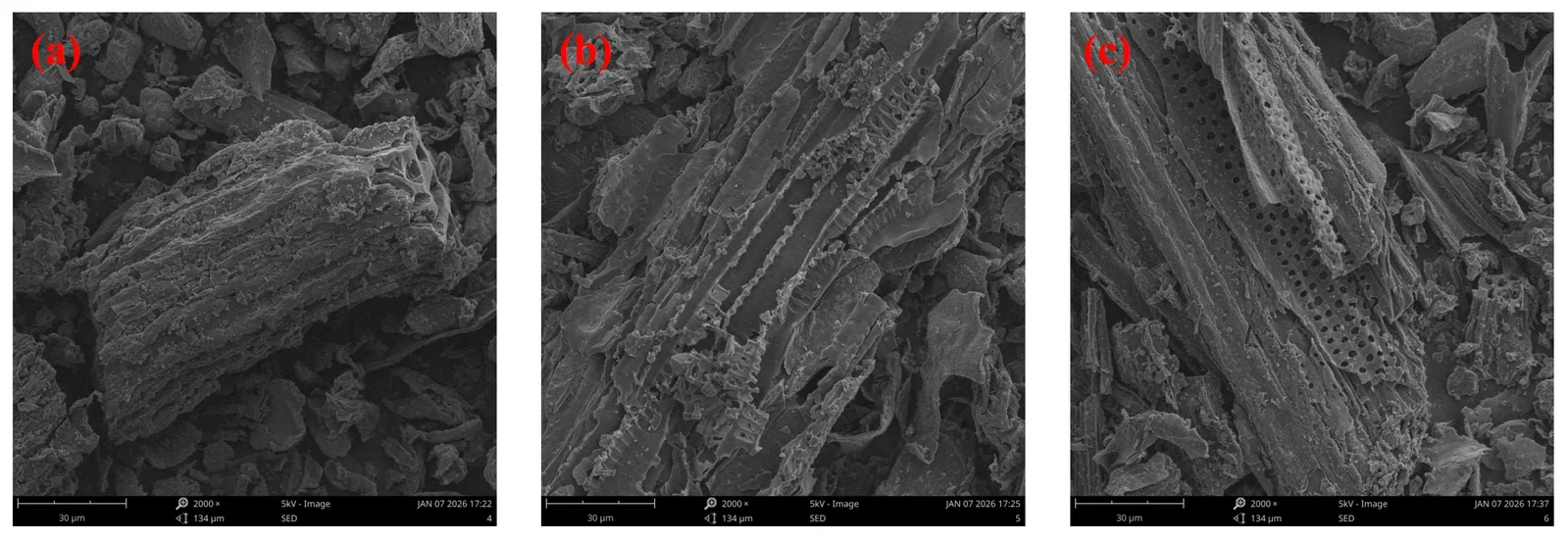
SEM images of *Artemisia*-derived biochars prepared at 400 °C (**a**), 500 °C (**b**), and 600 °C (**c**).

To investigate the influence of pyrolysis temperature on biochar surface morphology, SBC400, SBC500, and SBC600 were examined by SEM. As shown in Figure 1, under pyrolytic conditions the residual organic components of the biomass underwent pyrolytic gasification, and the escape of large amounts of organic volatiles brought about a marked transformation of the biochar microstructure [12]. At 400 °C, pores began to develop, and numerous micropores and mesopores with non-uniform diameters appeared on the surface, although connectivity between pores remained weak. The 500–600 °C range was a stage of full pore development: micropores gradually coalesced into mesopores and macropores to form an interconnected pore network, surface roughness increased substantially, and the carbon skeleton assumed a loose, layered structure. With a further rise in temperature, some of the larger carbon skeletons contracted and parts of the pore structure collapsed; nevertheless, the overall degree of pore development continued to increase [13]. SEM images showed differences in surface morphology among the three *Artemisia*-derived biochars. SBC400 exhibited relatively limited visible pore development, whereas SBC500 and SBC600 displayed more evident pores and a rougher, layered surface (Figure 1).

#### 3.1.3. Pore Structure

**Figure 2:**
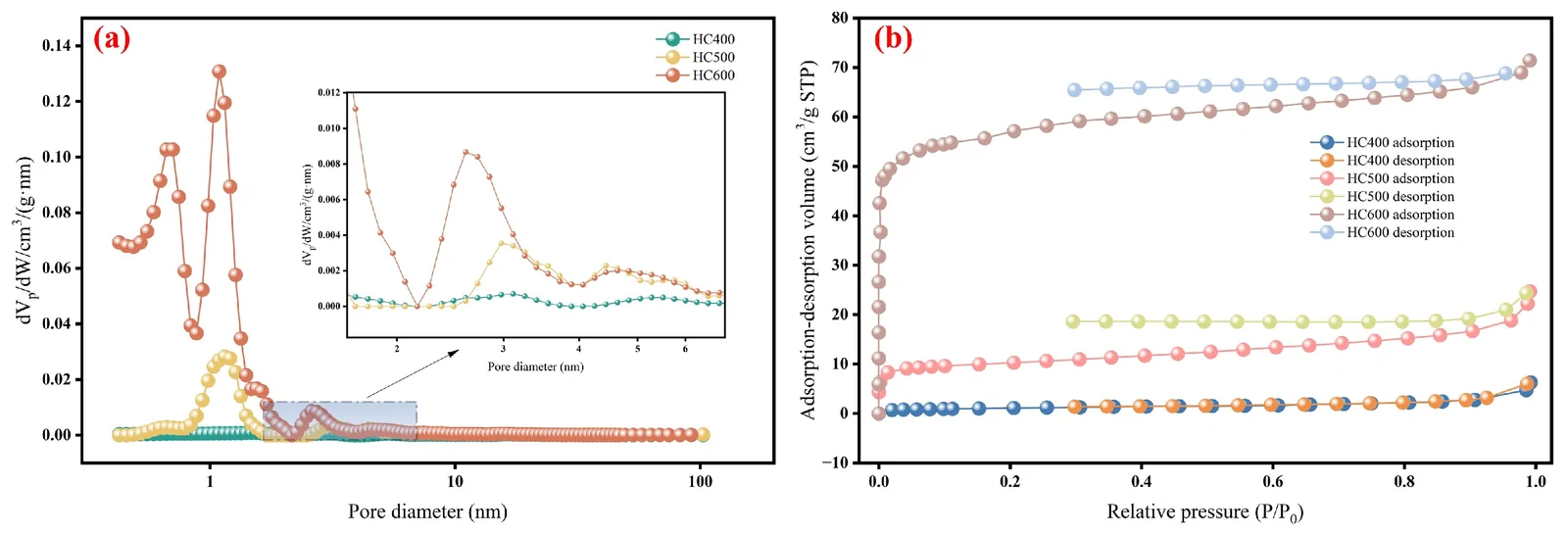
(**a**) Specific surface area adsorption–desorption curves of pyrolytic carbon. (**b**) Pore volume adsorption curves of pyrolytic carbon.

BET analysis showed marked differences in pore structure among the *Artemisia*-derived biochars prepared at different temperatures (Figure 2; Table 3). Specific surface area increased from 4.08 m^2^ g^−1^ in SBC400 to 38.53 m^2^ g^−1^ in SBC500 and 218.31 m^2^ g^−1^ in SBC600. Pore volume increased from 0.0089 to 0.0370 and 0.1102 cm^3^ g^−1^, respectively, whereas average pore diameter decreased from 8.7673 to 3.8452 and 2.0199 nm. The nitrogen adsorption–desorption profiles also differed among the samples: SBC400 showed relatively low adsorption, while SBC500 and SBC600 exhibited more pronounced adsorption and hysteresis. Together, these results indicate greater pore development at higher pyrolysis temperatures [14,15,16].

#### 3.1.4. Evolution of Surface Functional Groups

**Figure 3:**
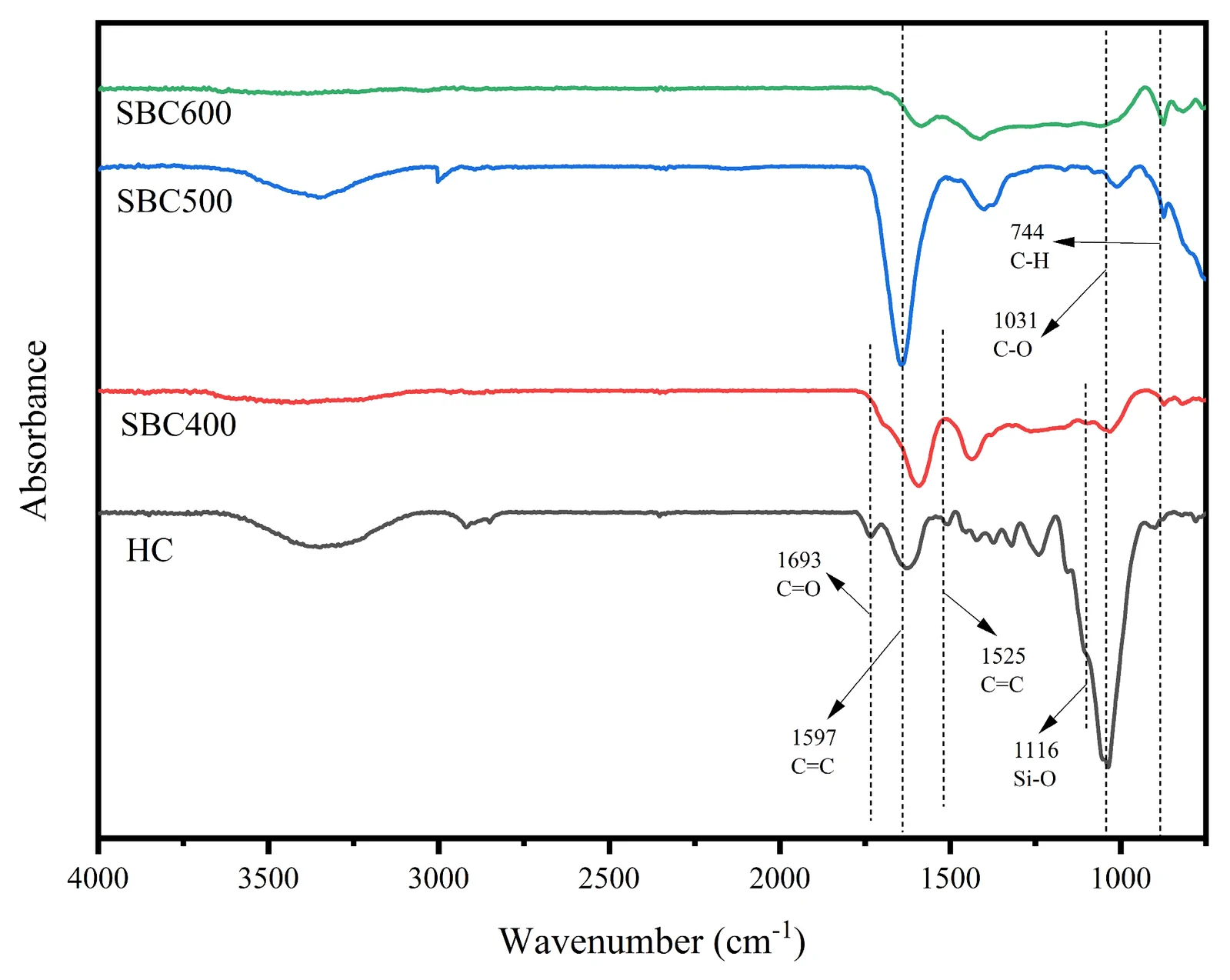
FTIR spectra of biochar before and after treatment.

FTIR spectra showed temperature-associated changes in the surface functional groups of the *Artemisia*-derived biochars in Figure 3. The aliphatic C–H band near 2926 cm^−1^ became negligible above 400 °C, whereas the aromatic C=C bands near 1587 and 1525 cm^−1^ became relatively more prominent [17]. The C–O band near 1031 cm^−1^ weakened with increasing pyrolysis temperature, while a broad O–H band was observed near 3350 cm^−1^. Overall, higher-temperature pyrolysis was associated with weaker aliphatic and C–O signals and more prominent aromatic features [18,19].

#### 3.1.5. Trace-Metal Contents and Their Response to Temperature

**Figure 4:**
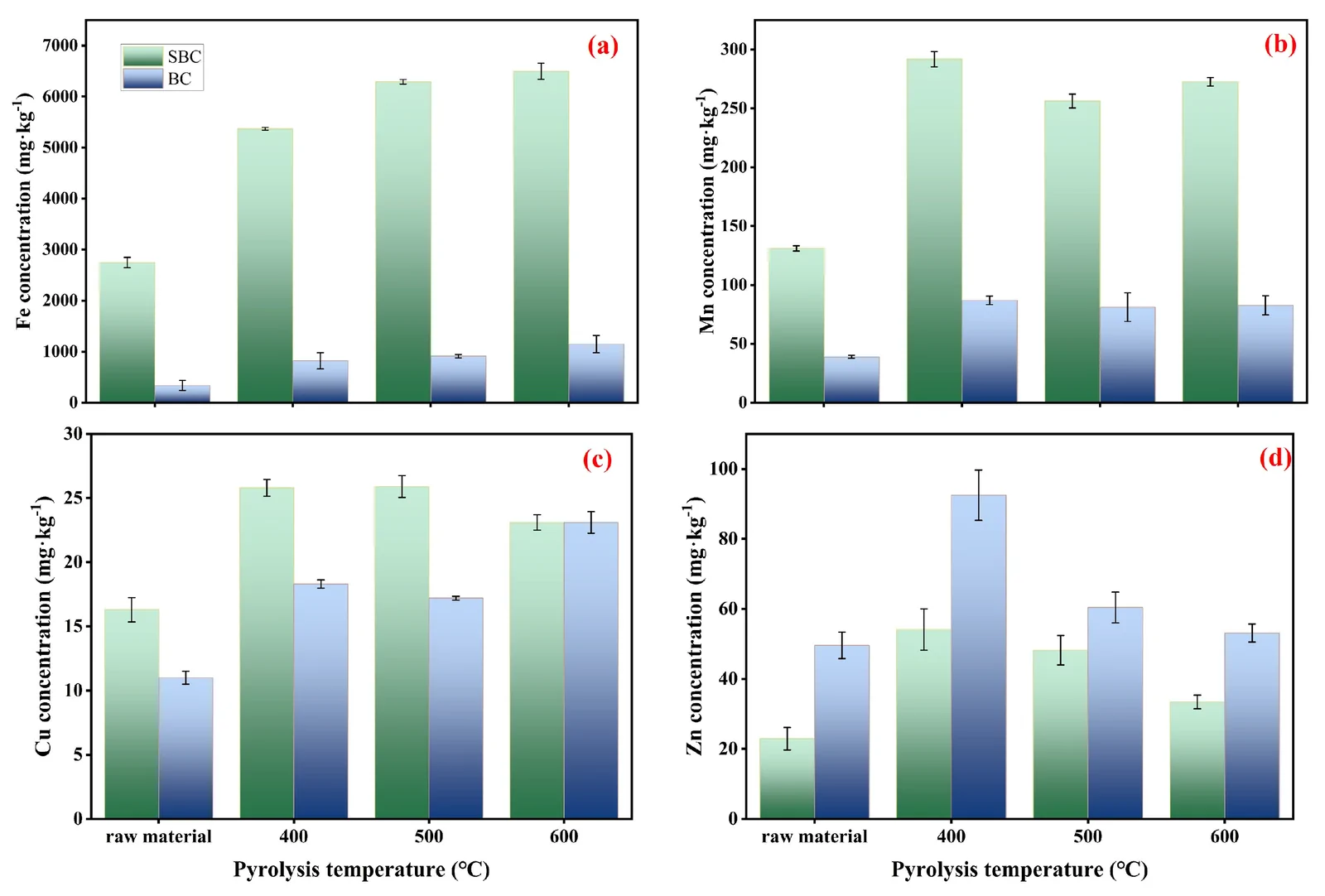
Microscopic metal element content in pyrolytic carbon prepared from two raw materials at different temperatures: (**a**) Fe content in pyrolytic carbon; (**b**) Mn content in pyrolytic carbon; (**c**) Cu content in pyrolytic carbon; (**d**) Zn content in pyrolytic carbon.

Elemental concentrations varied with feedstock and pyrolysis temperature (Figure 4). In *Artemisia*-derived biochar, Fe concentration increased from 5369.59 mg kg^−1^ in SBC400 to 6496.60 mg kg^−1^ in SBC600. Mn concentrations peaked at 400 °C and subsequently declined, while Zn concentrations also decreased at higher temperatures after reaching a maximum. Cu concentrations showed comparatively limited changes. Across the tested temperatures, *Artemisia*-derived biochars contained more Fe, Mn, and Cu than maize straw biochars, whereas Zn concentrations were higher in maize straw biochars. These concentration patterns indicate element-specific differences between the tested materials, but they do not independently establish elemental retention or volatilization because complete mass balance measurements, gas-phase measurements, and sufficiently detailed washing records were unavailable.

Because the two feedstocks differed little in their initial Cu contents, their Cu levels converged after high-temperature pyrolysis [20]. Zn content first rose and then fell, peaking at 400 °C and declining at 600 °C. At every temperature, BC contained more Zn than SBC, opposite to the pattern observed for the other three elements. A plausible interpretation is that Zn is less thermally stable than the other elements: ZnO has been reported to sublime and volatilize at high temperatures, so that at 600 °C, elemental loss could outweigh the concentration effect and produce the observed decline. Because direct gas-phase and mass balance measurements were not made, this interpretation is tentative rather than demonstrated. The consistently higher Zn in BC may also reflect the higher intrinsic Zn background of the maize straw feedstock.

### 3.2. Effects of Pyrolyzed Biochar on Ryegrass Growth

**Figure 5:**
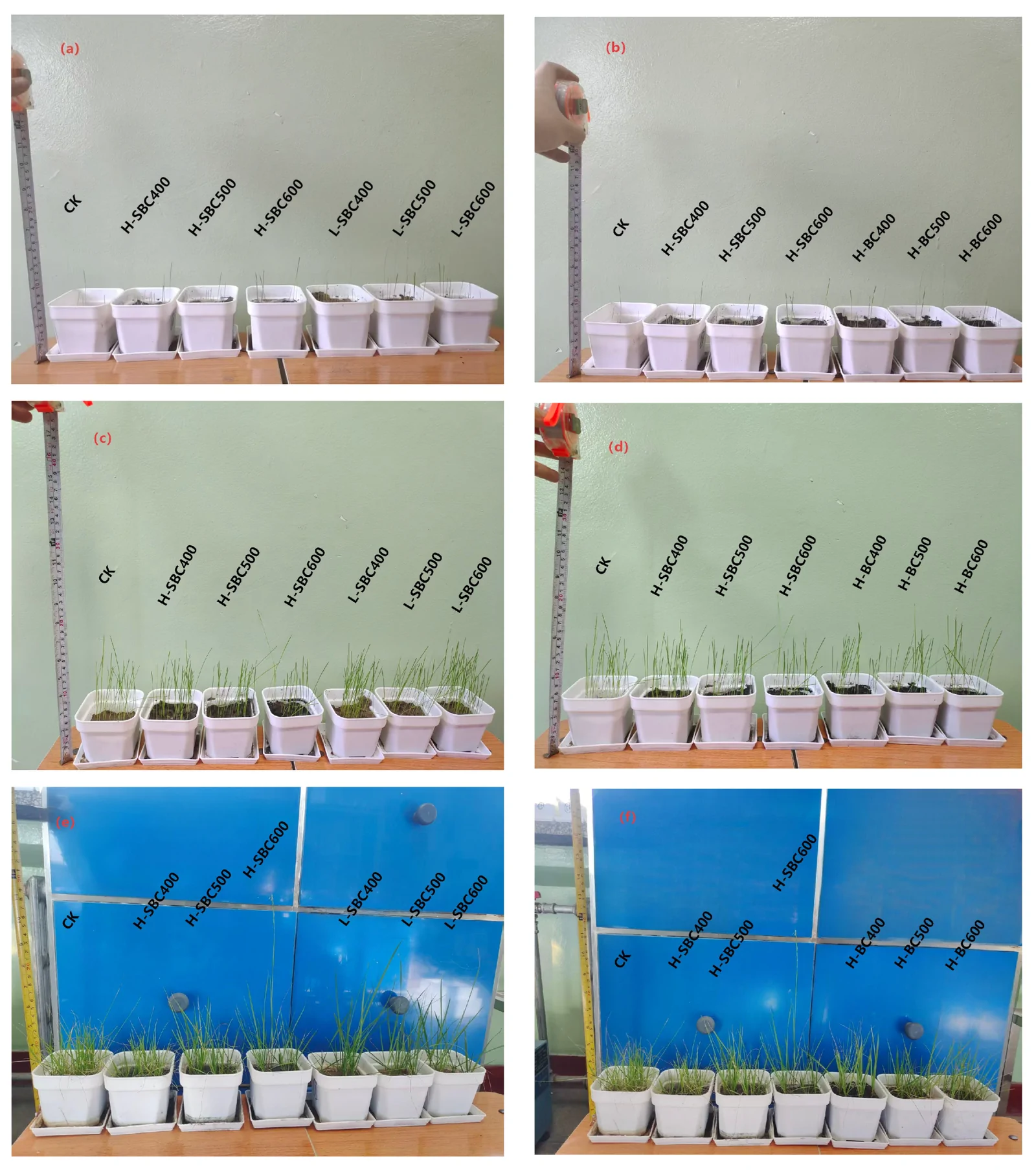
Control chart of ryegrass growth under different pyrolytic carbon treatment groups: (**a**) 7-day growth status of ryegrass with varying pyrolytic carbon application rates; (**b**) 7-day growth status of ryegrass treated with different raw material pyrolytic carbon; (**c**) 21-day growth status of ryegrass with different pyrolytic carbon application rates; (**d**) 21-day growth status of ryegrass treated with different raw material pyrolytic carbon; (**e**) 35-day growth status of ryegrass with varying pyrolytic carbon application rates; (**f**) 35-day growth status of ryegrass treated with different raw material pyrolytic carbon.

Ryegrass growth under the biochar treatments is shown in Figure 5 and Table 4. After biochar addition, plant height and root length were strongly influenced by pyrolysis temperature and application rate, whereas feedstock type exerted only a weak effect. Both indices increased continuously with pyrolysis temperature. At the low application rate, plant height and root length were 12.7 and 6.6 cm under L-SBC400, and 13.2 and 8.3 cm under L-SBC600, representing increases of 3.94% and 25.76% over the 400 °C treatment; the BC group showed exactly the same trend, with BC600 exceeding BC400 by 3.20% in plant height and 20.00% in root length. Biochar application rate also had a pronounced effect on ryegrass growth. At the low rate of 1%, biochars produced at all temperatures raised plant height by 52.44–60.98% and root length by 12.07–43.10% relative to CK. At the high rate of 5%, the corresponding increases were only 3.66–12.20% and 1.72–24.14%, indicating a marked attenuation of the growth-promoting effect. At the 5% application rate, several treatments showed higher soil pH at harvest, but the temporal dynamics of pH and its causal contribution to plant responses were not determined. At the same temperature and application rate, SBC consistently outperformed BC in growth promotion, although the difference in plant height between L-SBC600 and L-BC600 was small. At the 1% application rate, plant height and root length were generally higher in the SBC treatments than in the corresponding BC treatments. However, the difference in plant height between L-SBC600 and L-BC600 was small (Figure 5; Table 4).

### 3.3. Effects of Pyrolyzed Biochar on Chlorophyll Content of Ryegrass

**Figure 6:**
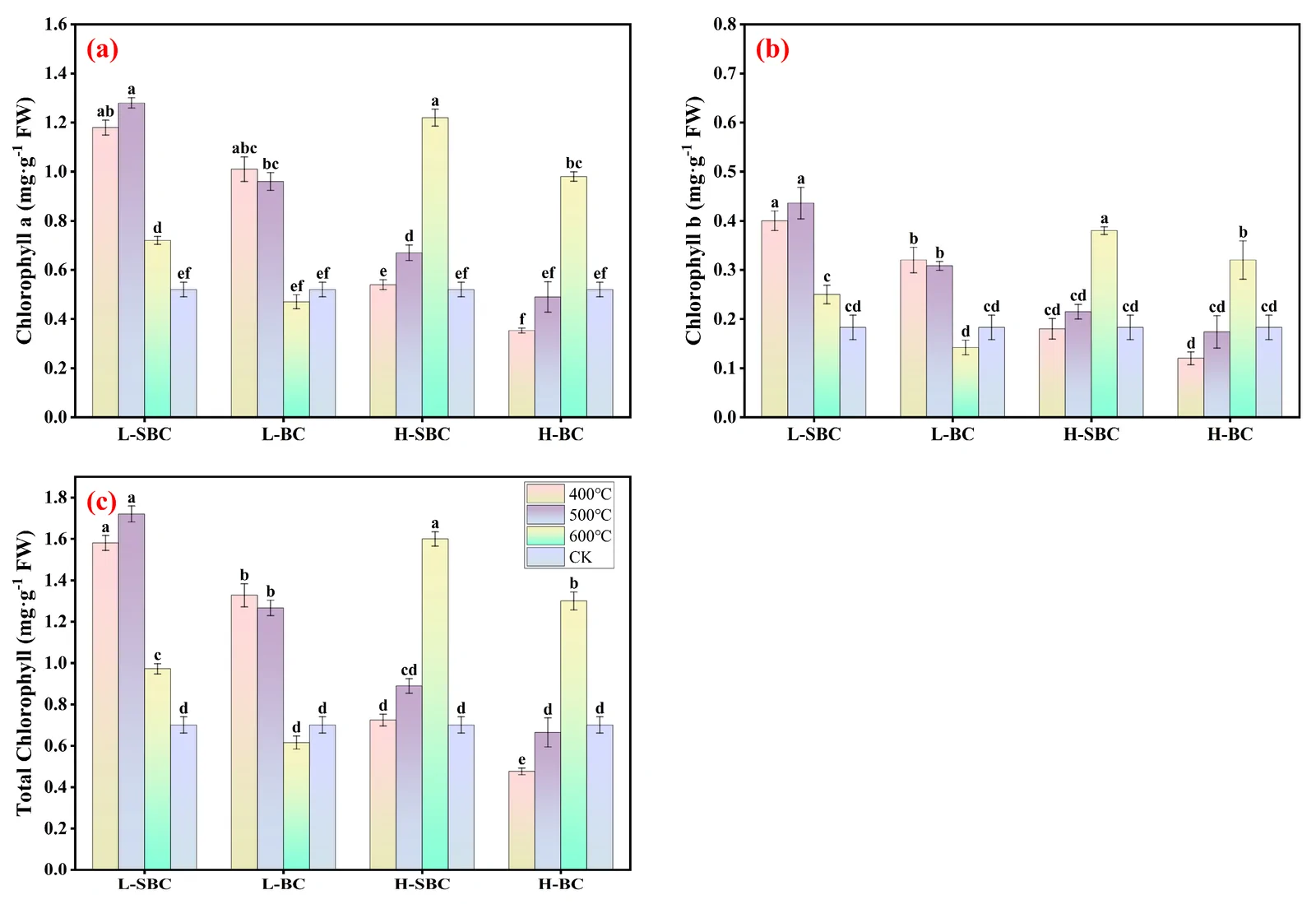
Effects of different pyrolytic carbon additions on chlorophyll a (**a**), chlorophyll b (**b**), and total chlorophyll (**c**) content in ryegrass leaves (Note: different lowercase letters indicate significant differences among all groups, 12 treatments and CK, by one-way ANOVA followed by Tukey’s HSD test; means sharing letter do not differ at *p* < 0.05).

Chlorophyll responses varied with application rate [21], pyrolysis temperature, and feedstock (Figure 6). At the 1% application rate, total chlorophyll increased with temperature from 400 to 500 °C and then decreased at 600 °C. L-SBC500 had the highest total chlorophyll content, reaching 1.72 mg g^−1^, which was 145.71% higher than in CK. At the 5% application rate, chlorophyll content increased with pyrolysis temperature, and only H-SBC600 and H-BC600 showed significantly higher chlorophyll contents than CK; the 400 and 500 °C treatments did not differ significantly from CK. At 500 °C and the 1% application rate, total chlorophyll in L-SBC500 was 35.86% higher than in L-BC500, with a significant difference (*p* < 0.05). Because the photosynthetic rate was not measured, these results indicate changes in chlorophyll content rather than direct changes in photosynthetic performance.

### 3.4. Effects of Pyrolyzed Biochar on Antioxidant Enzyme System of Ryegrass

**Figure 7:**
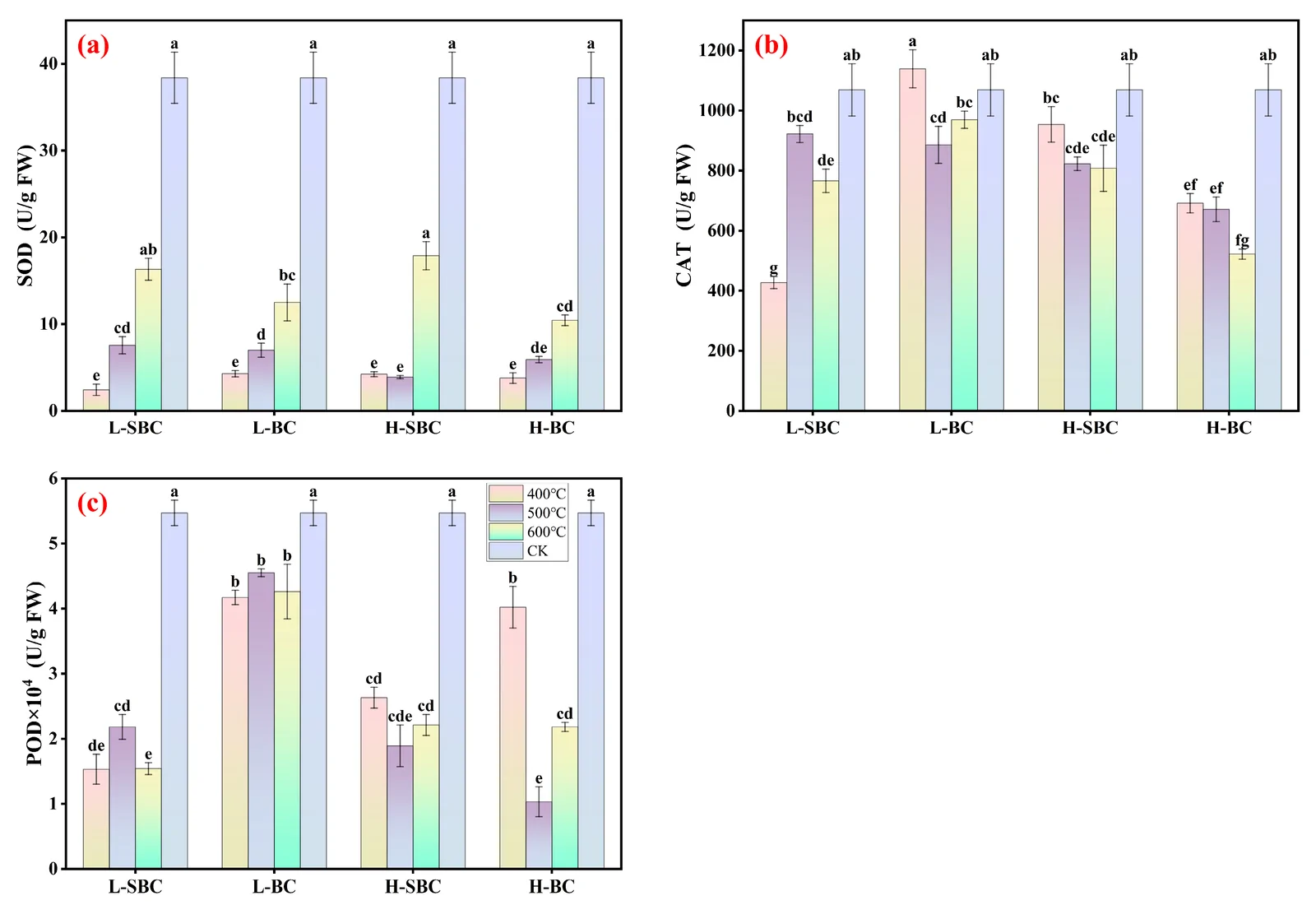
Effects of different pyrolytic carbon additions on SOD (**a**), CAT (**b**), and POD (**c**) content in ryegrass (Note: different lowercase letters indicate significant differences among all groups, 12 treatments and CK, by one-way ANOVA followed by Tukey’s HSD test; means sharing letter do not differ at *p* < 0.05).

Biochar treatments altered SOD, CAT, and POD activities in ryegrass leaves (Figure 7). Enzyme activities were generally lower in the 1% treatments than in CK and the corresponding 5% treatments. In L-SBC400, SOD and CAT activities were 2.44 and 427.14 U g^−1^, respectively, representing decreases of 93.65% and 60.04% relative to CK. Under the 1% application rate at 400 °C, CAT and POD activities in the *Artemisia*-derived biochar treatment were 62.50% and 63.31% lower, respectively, than in the corresponding maize straw biochar treatment. These results demonstrate treatment-dependent changes in antioxidant enzyme activity. However, because reactive oxygen species accumulation and lipid peroxidation were not measured, lower enzyme activities alone cannot establish reduced oxidative damage or improved stress tolerance.

### 3.5. Migration and Allocation of Trace Elements in Soil–Ryegrass System

Fe, Mn, Cu, and Zn are micronutrients essential to ryegrass growth and development and participate directly in key physiological processes, including chlorophyll synthesis [22,23,24], antioxidant enzyme formation [25,26], and photosynthetic electron transport [27]. Plant tissue concentrations describe elemental concentration patterns but cannot quantify total elemental uptake without corresponding plant biomass measurements. We measured Fe, Mn, Cu, and Zn concentrations in ryegrass shoots and roots and calculated the corresponding translocation factors to compare treatment responses.

#### 3.5.1. Variations in Trace-Metal Contents in Ryegrass Shoots and Roots

**Figure 8:**
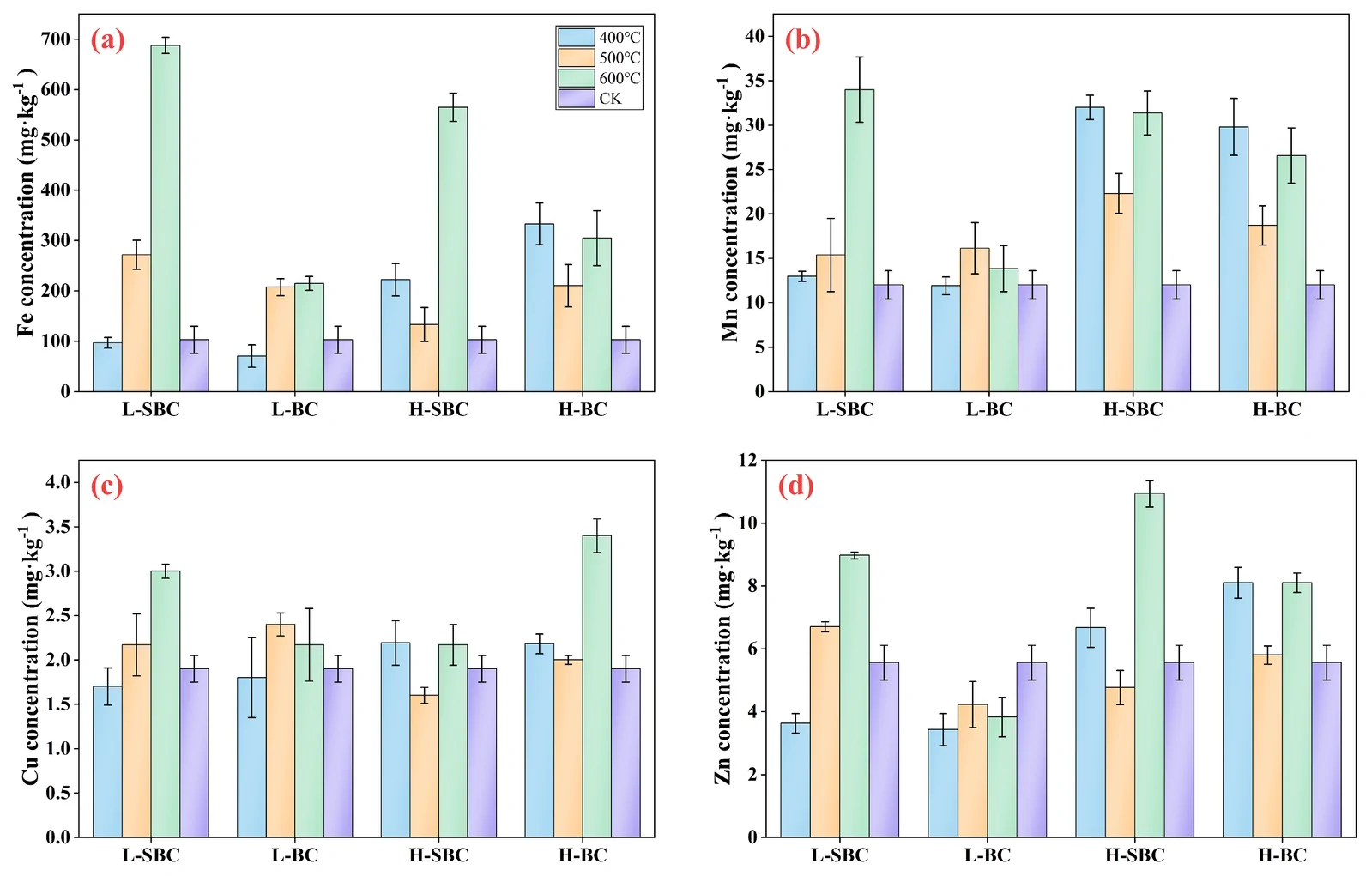
Effects of biochar treatments on Fe (**a**), Mn (**b**), Cu (**c**), and Zn (**d**) concentrations in ryegrass shoots. Error bars represent standard deviation (SD) of three independent replicate pots (n = 3); Differences among all groups (12 treatments and CK) by one-way ANOVA followed by Tukey’s HSD test (*p* < 0.05).

**Figure 9:**
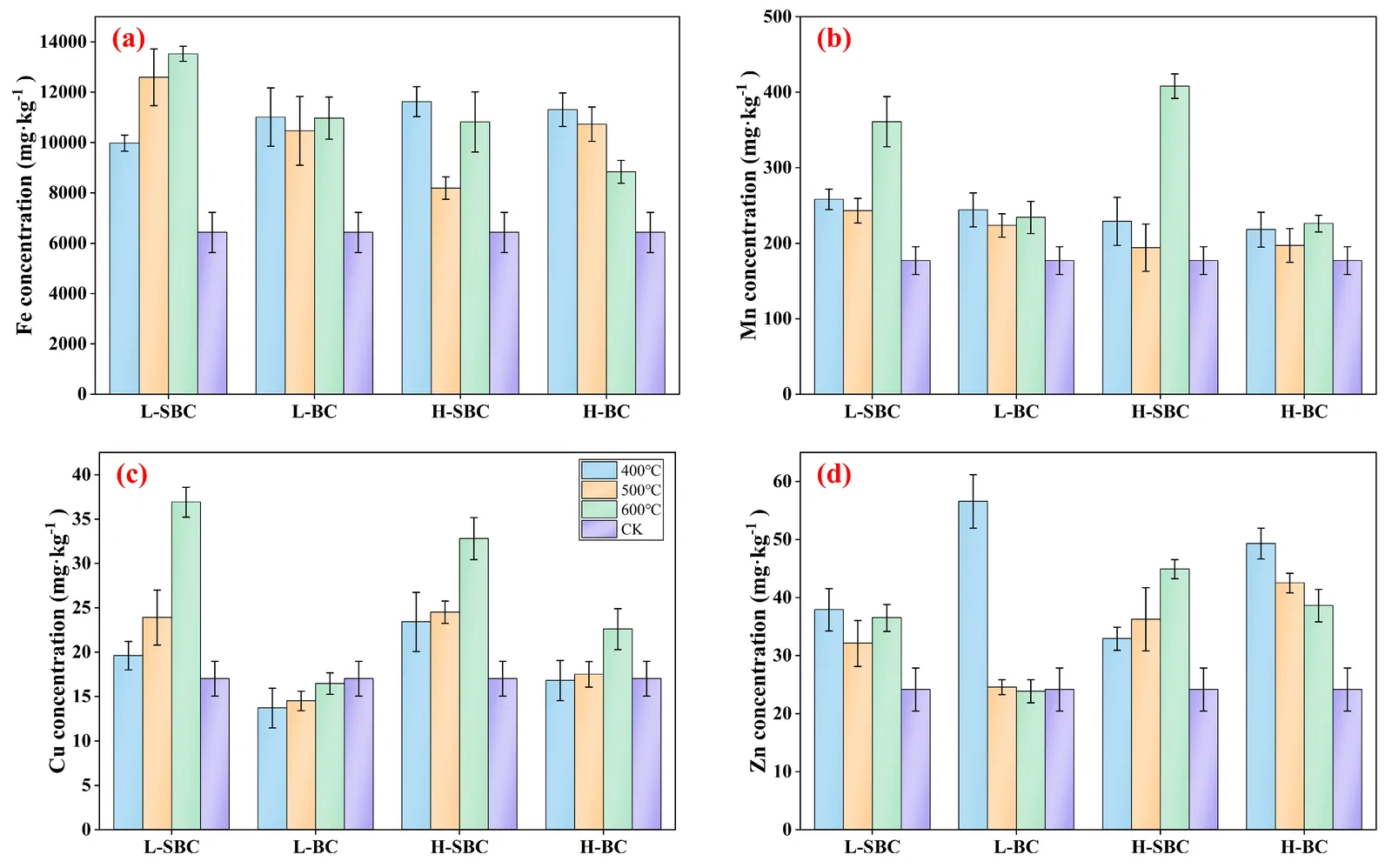
Effects of biochar treatments on Fe (**a**), Mn (**b**), Cu (**c**), and Zn (**d**) concentrations in ryegrass roots. Error bars represent standard deviation (SD) of three independent replicate pots (n = 3); Differences among all groups (12 treatments and CK) by one-way ANOVA followed by Tukey’s HSD test (*p* < 0.05).

Fe, Mn, Cu, and Zn concentrations in ryegrass shoots and roots varied among the biochar treatments (Figure 8 and Figure 9). Root concentrations were higher than shoot concentrations across the treatments, with the largest root–shoot difference observed for Fe [27,28]. In shoots, L-SBC600 had Fe and Mn concentrations of 687.79 and 34.22 mg kg^−1^, respectively; shoot Zn was highest in H-SBC600 at 10.93 mg kg^−1^, whereas shoot Cu was highest in H-BC600 at 3.40 mg kg^−1^. The 5% application rate produced significantly higher shoot Mn and Zn concentrations than the 1% rate under the reported comparisons. Shoot Fe concentration in L-SBC500 was 31.08% higher than in L-BC500.

In roots, Fe and Mn concentrations under L-SBC600 reached 13,526.91 and 361.25 mg kg^−1^, respectively, corresponding to 19.67 and 10.62 times their concentrations in shoots. Under the low application rate, root Fe and Cu generally increased with pyrolysis temperature, whereas root Mn and Zn showed non-monotonic responses. Root concentrations of several elements were generally higher in *Artemisia*-derived biochar treatments than in corresponding maize straw biochar treatments. Because root surface-associated elements were not distinguished from internally absorbed elements, and plant biomass was not used to calculate total uptake, these concentration data cannot quantify total elemental uptake or establish transfer of biochar-derived elements into ryegrass.

#### 3.5.2. Analysis of Trace-Metal Translocation Factors

Translocation factors (TFs), calculated as shoot-to-root concentration ratios, varied among elements and treatment combinations. Zn generally showed the highest TF, whereas Fe showed the lowest. The Zn TF in L-SBC600 reached 0.246. TF describes relative concentration partitioning between shoots and roots rather than a direct transport rate. Because root-surface-associated elements were not distinguished from internally absorbed elements, the TF values should be interpreted cautiously. Because the shoot and root concentrations are measured on the same basis, any common reporting-basis factor cancels in the ratio; so, the TF values are independent of the fresh-/dry-weight basis.

### 3.6. Effects of Pyrolyzed Biochar on Soil Chemical Properties and Enzyme Activities

Biochar treatments produced different responses in soil pH, soil organic carbon (SOC), urease activity, and alkaline phosphatase activity (Table 5). Soil pH ranged from 8.85 to 9.53 among the biochar treatments, compared with 8.97 in CK. The highest pH, 9.53, was recorded in H-SBC500. SOC increased from 7.43 g kg^−1^ in CK to 8.30–46.50 g kg^−1^ across the biochar treatments, with the highest value in H-SBC400. These end-point values include carbon added with the biochar as well as root-derived carbon accumulated during cultivation; the interpretation of the SOC change is discussed in Section 4.2.

Urease activity ranged from 99.8 to 172.3 U g^−1^ among the biochar treatments, compared with 71.9 U g^−1^ in CK. H-SBC400 had the highest activity, at 172.3 U g^−1^, representing an increase of 139.64% relative to CK. Alkaline phosphatase activity ranged from 1277 to 1721 U g^−1^ among the biochar treatments, compared with 726 U g^−1^ in CK. L-SBC400 had the highest activity, at 1721 U g^−1^, representing an increase of 137.05% relative to CK. Under the 5% application rate, alkaline phosphatase activity increased with pyrolysis temperature within both feedstock series. Overall, the results indicate that the responses of soil chemical properties and enzyme activities depended on biochar feedstock, pyrolysis temperature, and application rate.

### 3.7. Effects of Pyrolyzed Biochar on Soil Available Element Contents

Soil available Fe, Mn, Cu, and Zn are the nutrient forms that plants can directly absorb and utilize [28]; their contents directly determine the trace-element supply capacity of alkaline infertile soil and constitute the key link between biochar application and plant nutrient uptake [29,30].

**Figure 10:**
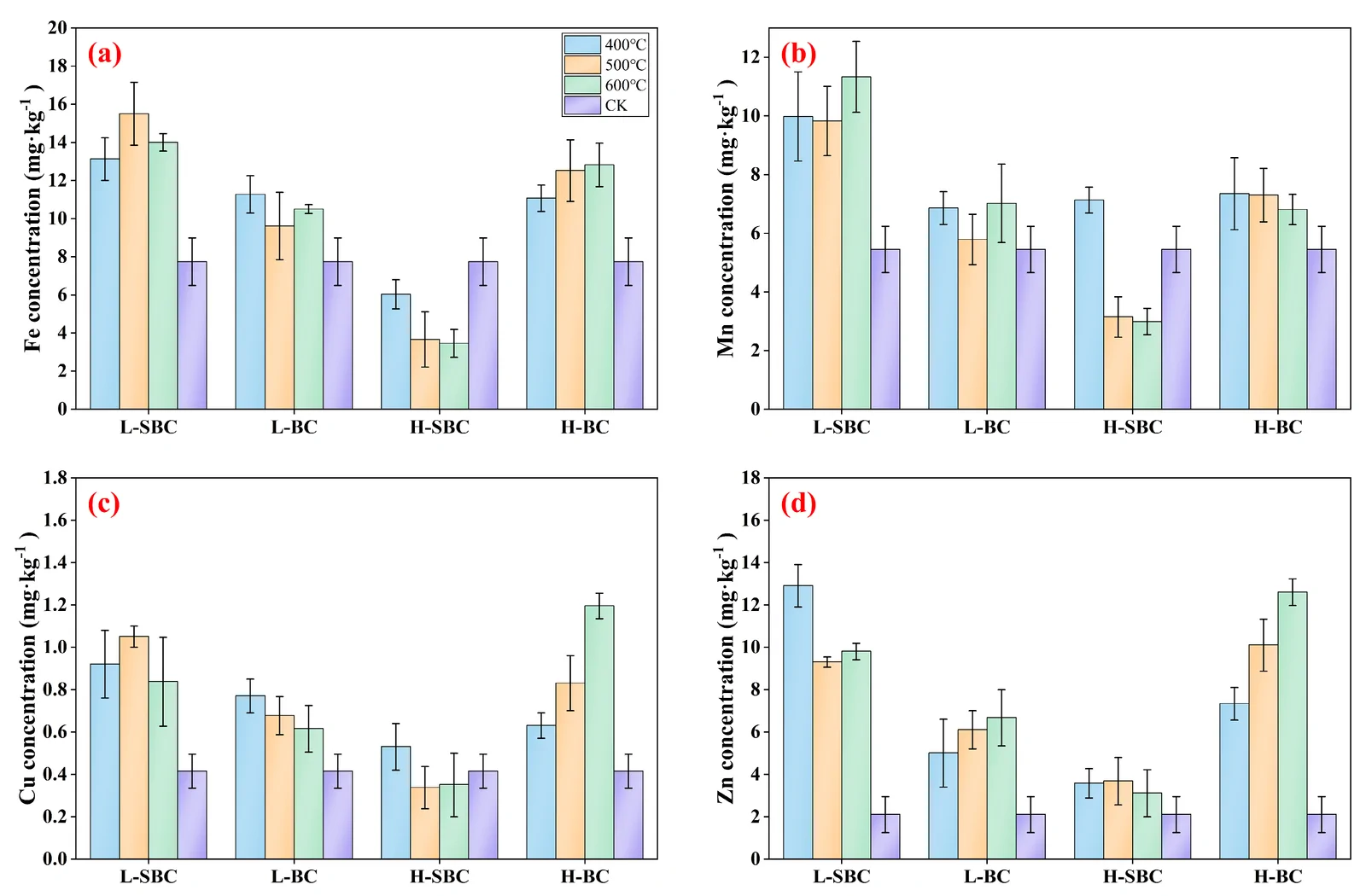
Effects of biochar treatments on DTPA-extractable Fe (**a**), Mn (**b**), Cu (**c**), and Zn (**d**) concentrations in soil. Error bars represent standard deviation (SD) of three independent replicate pots (n = 3). Differences among all groups (12 treatments and CK) by one-way ANOVA followed by Tukey’s HSD test (*p* < 0.05).

The effects of biochar treatments on DTPA-extractable Fe, Mn, Cu, and Zn are shown in Figure 10. At the 1% application rate, L-SBC500 exhibited DTPA-extractable Fe, Mn, Cu, and Zn concentrations of 15.50, 9.83, 1.05, and 9.30 mg kg^−1^, respectively, corresponding to increases of 100.26%, 80.37%, 153.01%, and 342.86% relative to CK. For *Artemisia*-derived biochar, the high-rate treatments generally showed lower DTPA-extractable micronutrient concentrations than the corresponding low-rate treatments, whereas maize straw biochar showed the opposite pattern in several comparisons. These results demonstrate treatment-associated changes in the extractable micronutrient pool. Notably, Cu and Zn in the H-BC groups rose rather than fell with increasing temperature, probably because the more developed pore structure of the 600 °C biochar shifts the metal-ion adsorption–desorption equilibrium toward release of the available forms [31].

## 4. Discussion

Throughout this discussion, the proposed mechanisms are offered as interpretations consistent with the measured data; because elemental speciation, volatilization and gas-phase losses, and the time course of soil pH were not measured directly, they are not presented as demonstrated processes.

### 4.1. Pyrolysis Temperature and Biochar Properties

Pyrolysis temperature altered biochar morphology, pore structure, surface functional groups, and measured elemental concentrations. SEM, BET, and FTIR results characterize these structural and chemical changes but do not directly establish elemental speciation, volatilization pathways, or release kinetics. Although the increased Fe concentrations are consistent with concentration effects caused by organic matter loss, the estimated apparent retention indicates that complete Fe retention cannot be assumed. Likewise, the observed declines in Mn and Zn concentrations do not independently demonstrate volatilization of MnO or ZnO [20]. These mechanisms remain plausible explanations requiring direct verification through elemental mass balance analysis, speciation characterization, and measurements of element release or gaseous losses.

### 4.2. Application-Rate Effects

Application rate is a key variable determining the soil-ameliorating performance of pyrolyzed biochar [32,33], and the contrasting outcomes at 1% and 5% reveal a dual mechanism. At the low rate, several plant growth and soil enzyme indicators responded favorably, although the underlying mechanisms and the contribution of biochar-derived carbon were not independently quantified. At the high rate, a plausible interpretation is that the large input of alkaline constituents imposed an alkaline shock: soil pH at harvest was as high as 9.53, which could have promoted hydroxide precipitation and immobilization of trace elements [34,35] and may have suppressed pH-sensitive enzymes such as alkaline phosphatase, thereby lowering nutrient bioavailability [36]. Because soil pH was measured only at harvest and hydroxide precipitation was not directly determined, this sequence is a proposed mechanism consistent with the observed pattern rather than a demonstrated causal chain. The tendency of high-temperature biochars to retain a beneficial effect at the high rate may be related to their more stable carbon skeleton and slower release of alkaline constituents [32,37], although this was likewise not measured directly. These results indicate that application rate is a critical determinant of biochar performance in alkaline soil. Under the present 40-day pot conditions, the 1% rate produced more favorable responses for several plant growth and micronutrient-related indicators, whereas the 5% rate increased some soil enzyme activities and plant element concentrations.

Interpretation of the soil organic carbon (SOC) values requires care because SOC is a measured, dynamic pool rather than the amount of carbon added with the biochar. The increase in SOC relative to CK cannot be compared one-to-one with the carbon supplied by the biochar (the maximum estimated from the non-ash fraction), because the harvested soil also contains plant-derived carbon that accumulated during the 40-day experiment. First, the biochar treatments, which produced more vigorous root systems with root length up to 43.10% greater at the 1% rate, supplied more root biomass, root exudates, and sloughed rhizodeposits than CK; this photosynthetically fixed carbon is measured as SOC but did not originate from the biochar. Second, biochar can exert a negative priming effect by sorbing organic molecules and protecting them from microbial mineralization, so that more of the native soil organic carbon is retained in the amended pots than in CK. Third, the dichromate external heating method (NY/T 1121.6-2006) provides a method-defined estimate that includes both biochar-derived and plant-derived carbon and applies the standard oxidation correction factor. Together, these processes explain why the measured SOC gain can exceed the simple biochar-carbon input without any inconsistency in the data. The SOC values are therefore reported as direct end-point measurements and are not used to claim that the entire increase is sequestered biochar carbon; a closed carbon mass balance would require separate measurements of root biomass and soil CO_2_ efflux, which are outside the scope of this short-term experiment.

### 4.3. Feedstock-Related Differences

The elemental profiles of the two biochars differed, with *Artemisia*-derived biochar containing higher concentrations of Fe, Mn, and Cu, whereas maize straw biochar contained more Zn. These differences may be associated with the elemental composition of the source biomass. However, the two feedstocks were collected from different locations and were not obtained under a common cultivation or environmental setting. Consequently, the observed differences cannot be attributed exclusively to plant species. Furthermore, soil-extractable and plant tissue concentrations describe treatment-associated elemental patterns but do not quantify elemental transfer from biochar or total plant uptake. Direct release measurements, appropriate source attribution methods, and plant biomass data would be needed to establish these processes quantitatively.

### 4.4. Resource Utilization Potential and Environmental Risk Assessment

Taken together, the pot experiment showed that 1% *Artemisia*-derived biochar produced at 400–600 °C affected ryegrass growth, physiological traits, soil enzyme activities, and micronutrient availability, with the magnitude of the responses depending on pyrolysis temperature. SBC400 showed stronger effects on several soil enzyme and antioxidant response variables, whereas SBC500 and SBC600 were associated with greater responses in chlorophyll accumulation, plant growth, or micronutrient-related indices. These differences indicate that pyrolysis temperature modulates the functional characteristics of *Artemisia*-derived biochar, although direct micronutrient-release kinetics were not determined in the present study [38]. The environmental safety of biochar derived from mining-affected plant biomass nevertheless requires further assessment. Although previous studies have reported that pyrolysis can reduce the mobility or bioavailability of some metals, the present study did not directly quantify metal speciation or leaching behavior before and after pyrolysis. Moreover, long-term biochar aging may alter metal retention and release, depending on soil pH, redox conditions, and changes in the biochar matrix. The mobility and environmental risks of Cr, Cd, Pb, and Ni require further assessment because their extraction procedures and total input loads were not sufficiently documented in the present study. Future research should combine long-term field experiments with heavy-metal speciation analysis to establish an integrated whole-chain safe-utilization system encompassing feedstock screening, pyrolysis process optimization, and application rate control.

On a field scale, the application rates used here correspond, for a soil bulk density of 1.3 t m^−3^ and incorporation to 15 cm (about 1950 t of soil ha^−1^), to approximately 19.5 t biochar ha^−1^ at the 1% rate and 97.5 t ha^−1^ at the 5% rate (or 26 and 130 t ha^−1^ if incorporated to 20 cm). Because the 5% rate induced an alkaline shock, the practical recommendation is the low rate, on the order of 20 t ha^−1^. *Artemisia* biochar is not proposed as a commodity requiring a regional supply chain; it is intended as a local, on-site or near-site waste-to-resource route for biomass that would otherwise be stockpiled or landfilled, so the relevant quantity is the locally harvestable biomass rather than a regional production target. A quantitative regional harvest and supply assessment is beyond the scope of this proof-of-concept study and is identified as future work. The plant growth and micronutrient effects described here partly overlap with those reported for humic substances, which act through hormone-like signaling, complexation of cationic micronutrients, and changes in root morphology and rhizosphere chemistry [39,40]. We agree that humic substances are an effective organic amendment in high-pH soils. The two materials are nevertheless mechanistically distinct: humic substances are soluble/colloidal molecules that act directly on the root and in the soil solution, whereas biochar is a solid, porous matrix that acts largely indirectly through pH, sorption, water retention, and the provision of microbial habitat, and—in the present case—provides a route to valorize mining-area *Artemisia* biomass. The two are therefore complementary rather than interchangeable; this study does not claim that biochar is superior, and a direct comparison with humic substances is a logical subject for future work.

Several methodological limitations should be considered when interpreting the pot experiment results. Although the soil moisture was maintained at approximately 60% of the field water-holding capacity, moisture was not continuously logged; so, the effects of biochar-induced changes in water retention on the actual time course cannot be fully separated. In addition, although all established seedlings were retained, the final number of plants per pot was not recorded, which prevents a retrospective assessment of whether differences in plant density contributed to the observed responses. The plants within each pot were pooled and homogenized before the analytical subsample was taken, and the individual-plant growth measurements were averaged within the pot, yielding one independent observation per pot (n = 3). These uncertainties limit the extent to which the observed differences can be attributed exclusively to biochar treatment. Future experiments should record seedling emergence, survival, and final plant counts, continuously monitor soil moisture, and clearly define the aggregation of plant-level measurements at the pot level. Such improvements would strengthen the reproducibility of the experimental design and the reliability of comparisons among biochar treatments.

The main contribution of this study is a temperature- and application rate-resolved comparison of biochar produced from mining-affected *Artemisia* biomass with conventional maize straw biochar in an alkaline, low-fertility soil–ryegrass system. The results reveal a clear treatment-dependent pattern: 1% SBC600 produced the largest reported increases in plant height and root length, whereas 1% SBC500 produced the highest total chlorophyll content and was associated with substantial increases in DTPA-extractable soil micronutrients. In contrast, the 5% application rate increased soil pH to 9.53 and attenuated several plant and soil responses. These findings extend the evaluation of biochar feedstocks beyond conventional agricultural residues and demonstrate that the effects of mining-affected *Artemisia* biomass depend on pyrolysis temperature and application rate. Nevertheless, the present conclusions are limited to a 40-day pot experiment and concentration-based measurements. Direct measurements of elemental release, source attribution, speciation, and leaching, together with longer-term experiments, are required before broader agronomic application can be recommended.

## 5. Conclusions

Under the 40-day pot conditions, the effects of biochar on ryegrass and soil properties depended on feedstock, pyrolysis temperature, and application rate. At the 1% application rate, SBC600 increased plant height and root length by 60.98% and 43.10%, respectively, while SBC500 increased total chlorophyll by 145.71% relative to the control. The 5% application rate caused stronger soil alkalization and attenuated several plant and micronutrient-related responses, although some soil enzyme activities and plant element concentrations increased. *Artemisia*-derived biochar contained higher concentrations of Fe, Mn, and Cu than maize straw biochar, whereas Zn concentrations were higher in maize straw biochar. These findings indicate that mining-affected *Artemisia* biomass has potential for biochar production, but the present results do not establish the source or transfer of the measured elements or demonstrate long-term environmental safety. Future research should include independently replicated biochar production, longer-term pot and field experiments, and direct measurements of elemental mass balance, release, speciation, and leaching before broader agronomic application.

## Figures and Tables

**Table 1:** The initial physicochemical properties of the experimental soil before the pot experiment (mean values, n = 3).

Property	Value
Soil texture	Silt loam (sand 21.6%; silt 57.9%; clay 20.5%)
pH (soil/water, 1:2.5)	8.83
Electrical conductivity (1:5)	0.17 dS m^−1^
Soil organic carbon	7.18 g kg^−1^
Total nitrogen	0.53 g kg^−1^
Alkali-hydrolyzable nitrogen	35.2 mg kg^−1^
Available phosphorus	4.9 mg kg^−1^
Available potassium	121 mg kg^−1^
DTPA-extractable Fe	7.51 mg kg^−1^
DTPA-extractable Mn	5.38 mg kg^−1^
DTPA-extractable Cu	0.43 mg kg^−1^
DTPA-extractable Zn	2.16 mg kg^−1^

**Table 2:** Ash content and pH of *Artemisia*-derived biochars produced at different pyrolysis temperatures.

Biochar	Ash Content/%	pH
SBC400	52.4	10.04
SBC500	57.9	10.31
SBC600	66.3	10.09

**Table 3:** Analysis of specific surface area and porous structure of biochar.

Biochar	BET Specific Surface Area/m^2^ g^−1^	Pore Volume/cm^3^ g^−1^	Average Pore Diameter/nm
SBC400	4.08	0.0089	8.7673
SBC500	38.53	0.0370	3.8452
SBC600	218.31	0.1102	2.0199

**Table 4:** Plant height and root length of ryegrass under different biochar treatments.

Sample	Plant Height/cm	Root Length/cm
CK	8.2	5.8
L-SBC400	12.7	6.6
L-SBC500	13.2	7.4
L-SBC600	13.2	8.3
H-SBC400	8.6	6.1
H-SBC500	8.9	6.6
H-SBC600	9.1	6.8
L-BC400	12.5	6.5
L-BC500	13	7.3
L-BC600	12.9	7.8
H-BC400	8.5	5.9
H-BC500	8.6	6.3
H-BC600	9.2	7.2

Note: Values are treatment means (n = 3). The significance of the feedstock, pyrolysis temperature, and application rate effects and the treatment-versus-CK differences (Dunnett’s test, *p* < 0.05) are reported in the text (Section 3.2).

**Table 5:** Soil pH, soil organic carbon content, urease activity, and alkaline phosphatase activity under different biochar treatments.

Sample	pH	SOC (g/kg)	S-UE (U/g)	S-AKP/ALP (U/g)
CK	8.97	7.43	71.9	726
L-SBC400	9.26	21	132.3	1721
L-SBC500	9.07	9.48	124	1536
L-SBC600	8.92	8.3	109.6	1662
H-SBC400	9.39	46.5	172.3	1289
H-SBC500	9.53	39.3	159.5	1366
H-SBC600	9.39	34.48	135.9	1512
L-BC400	8.96	12	126.1	1428
L-BC500	8.85	11.2	120.2	1335
L-BC600	9.11	9.1	99.8	1327
H-BC400	8.91	33.96	149.2	1277
H-BC500	9.21	29.8	136.2	1296
H-BC600	9.12	33.1	106.1	1335

Note: Values are treatment means (n = 3). The significance of the feedstock, pyrolysis temperature, and application rate effects and the treatment-versus-CK differences (Dunnett’s test, *p* < 0.05) are reported in the text (Section 3.6).

## Data Availability

The original contributions presented in this study are included in the article. Further inquiries can be directed to the corresponding author.
